# Optical metrology embraces deep learning: keeping an open mind

**DOI:** 10.1038/s41377-022-00829-1

**Published:** 2022-05-17

**Authors:** Bing Pan

**Affiliations:** School of Aeronautic Science and Engineering, Beihang University, 100191 Beijing, China

**Keywords:** Imaging and sensing, Optical metrology

## Abstract

Optical metrology practitioners ought to embrace deep learning with an open mind, while devote continuing efforts to look for its theoretical groundwork and maintain an awareness of its limits.

Optical metrology is the science and technology concerning measurements using light. The development of physical sciences has been driven from the very beginning by optical metrology techniques. In return, optical metrology has been revolutionized by several major inventions of physical sciences, such as the laser, charged coupled device (CCD), and computer technology. Although optical metrology technologies have developed into problem-solving backbones in many science and engineering applications, they have already implemented the transition to their digital avatars for nearly half a century, entering an era of diminishing returns. After the three previous revolutions brought about by the laser, imaging sensor, and digital computing, which technology will reinvigorate optical metrology?

Deep learning is a type of machine learning that uses artificial neural networks to learn a mapping between input and output data^1^. Once trained, these models can predict outputs from supplied input data. In 2016, AlphaGo beating Lee Sedol, the best human player at Go, four matches to one was a truly seminal event in the history of machine learning and deep learning. Since then, we have witnessed its explosive growing and extensive applications in solving many tasks in computer vision, computational imaging, and computer-aided diagnosis^2^. In light of the tremendous success of deep learning in these related fields, researchers in optical metrology were unable to hold back their curiosities with regards to adopting this technology to further push the limits of optical metrology and provide new solutions in order to meet the upcoming challenges in the perpetual pursuit of higher accuracy, sensitivity, repeatability, efficiency, speed, and robustness.

The research group led by Prof. C. Zuo at Nanjing University of Science and Technology is a pioneer in introducing deep learning to optical metrology with a particular focus on fringe pattern analysis and fringe projection profilometry. In 2019, they developed a deep-learning-based fringe pattern analysis technique capable of combining the single-frame strength of spatial phase demodulation techniques with the high measurement accuracy of multi-frame phase-shifting techniques^3^. The network is trained on single fringe pattern matched with the label phase (ground-truth) reconstructed by the standard 12-step phase-shifting algorithm of the same sample. After training based on extensive dataset, the neural network can transform a single fringe pattern into an accurate phase map from that almost reproduces the result of the 12-step phase-shifting method, which is an astonishing feat for the field. Subsequentially, researchers in optical metrology started actively tilling this fertile field, as evidenced by the ever-increasing number of publications. Within a few short years, deep learning has been applied to various tasks of optical metrology, such as fringe denoising^4^, phase unwrapping^5,6^, and single-shot profilometry^7,8^.

In a recent issue of *Light*: *Science & Applications*, Zuo et al.^9^ presented a comprehensive review in the field of deep learning applied to optical metrology. They start from conventional methods and typical signal processing tasks in optical metrology, and then introduce the idea of data-driven evaluation with deep learning. As a smooth transition between the old and the new, they provided a brief introduction to deep learning and summarized the threefold advantages of its application to optical metrology: from “physics-model-driven” to “data-driven”, from “divide-and-conquer” to “end-to-end learning”, and from “solving ill-posed inverse problems” to “learning pseudo-inverse mapping”. Then a comprehensive overview where deep learning has already infiltrated almost every aspect of image processing tasks is presented, suggesting a paradigm shift from physics-based modeling to data-driven learning in optical metrology. The panoramic comparative picture reveals that using problem-specific deep learning models outperforms conventional knowledge or physical model-based approaches in most cases, especially for many optical metrology tasks whose physical models are complicated and acquired information is limited.

While promising, in many cases pretty impressive, results have been documented in the literature, Zuo et al. admitted that these works still represent early days in the application of deep learning to optical metrology. It is sensible to maintain a clear head and recognize that deep learning is not magic: it is essentially the process of using computers to help us find patterns within data. Since the information cannot “born out of nothing”, deep neural networks are usually pretty brittle, i.e., if we do not feed in the RIGHT kind of data in the RIGHT kind of format using the RIGHT kind of network model and training algorithm, we will get poor results.

In many applications of computer vision, people are always happy when the result looks good and realistic, no matter whether it is interpretable and quantifiable or not. However, adhering to the famous creed by Galileo: “Measure what is measurable, and make measurable when it is so”, practitioners in optical metrology is both open-minded and rigorous. In optical metrology, it is not only necessary to get a good-looking result, but also need to make sure that the result is accurate, reliable, repeatable, and traceable. Though we hope that such deep learning approaches always have a provably correct solution, no one can guarantee, at least not yet. Another well-known disadvantage of deep neural networks is their “black box” nature. Simply put, we do not know how or why the network came up with a certain output (Fig. 1). However, the interpretability is critical to optical metrology, as it allows us trust the methodology and understand the causes of mistakes. Shall we accept deep learning as the key problem-solving tool? Or should we reject such a black-box solution? These are controversial issues in the optical metrology community nowadays.

**Fig. 1:**
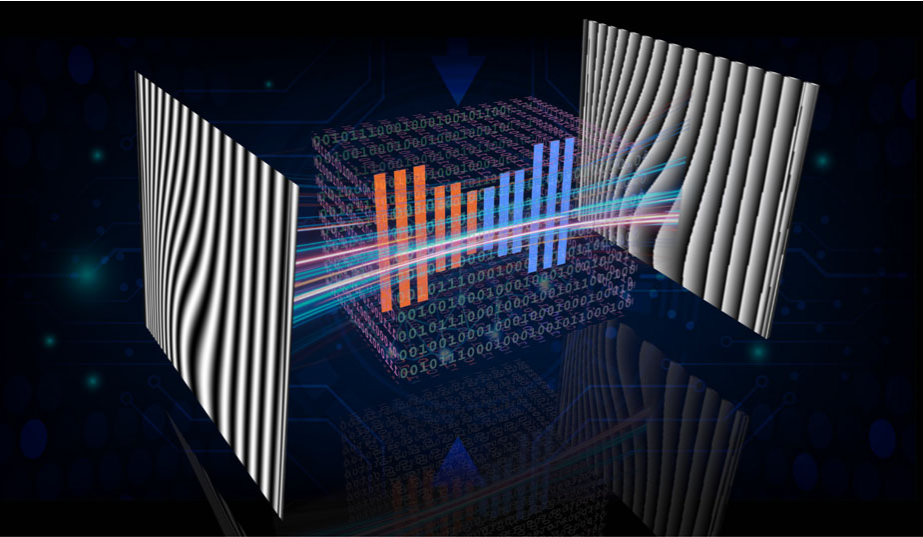
The pros and cons of applying deep learning to optical metrology. In deep-learning-based fringe analysis, a well-trained neural network can transform a single fringe pattern into an accurate phase map from that almost reproduces the result of the multi-step phase-shifting method, which is an astonishing feat for the field. But its internal mechanism tends to be very difficult to explain (“Black Box problem”)

Conjuring more from less must pay a price. There are still significant challenges in deep learning-based optical metrology: First, for model training, we need to acquire large amounts of experimental data with labels, which, even if available, is laborious and requires professional experts. Second, we need to look for the theoretical groundwork that would clearly explain the ways to define the optimal network structure and comprehend the reasons for its success or failure. Third, we should recognize that the power of deep learning approaches often comes at the expense of generalization (the ability to deal with never-before-experienced situations), i.e., their performance can be system, environment, and even sample dependent. Nevertheless, the progress of science comes from the continuous exploration to solve the unknown. So, I encourage optical metrology practitioners to embrace deep learning with an open mind while maintain an awareness of its limits.

All in all, nothing in deep learning-based optical metrology is to be feared. It is only to be understood and quantified. Recent research into Bayesian deep learning promises to assess the reliability of the network by explicitly quantifying uncertainty, which provides us an additional choice between “trusting the network without doubts” and “denying it completely”, namely “trusting it conditionally”^10^. As the emerging field slowly matures, deep learning is expected to graduate from black-box empirical representations to full-blown theoretical foundations, with a more profound impact not only on optical metrology, but also on optics and photonics as a whole.
